# Modeling the Impact of Climate Change on Cervid Chronic Wasting Disease in Semi-Arid South Texas

**DOI:** 10.3389/fepid.2022.889280

**Published:** 2022-05-26

**Authors:** Md Rafiul Islam, Ummugul Bulut, Teresa Patricia Feria-Arroyo, Michael G. Tyshenko, Tamer Oraby

**Affiliations:** ^1^Department of Mathematics, Iowa State University, Ames, IA, United States; ^2^Department of Mathematical, Physical, and Engineering Sciences, Texas A&M University-San Antonio, San Antonio, TX, United States; ^3^Department of Biology, The University of Texas Rio Grande Valley, Edinburg, TX, United States; ^4^Risk Sciences International, Ottawa, ON, Canada; ^5^School of Mathematical and Statistical Sciences, The University of Texas Rio Grande Valley, Edinburg, TX, United States

**Keywords:** chronic wasting disease, prion, climate change, cervids, carrying capacity, South Texas

## Abstract

Chronic wasting disease (CWD) is a spongiform encephalopathy disease caused by the transmission of infectious prion agents. CWD is a fatal disease that affects wild and farmed cervids in North America with few cases reported overseas. Social interaction of cervids, feeding practices by wildlife keepers and climate effects on the environmental carrying capacity all can affect CWD transmission in deer. Wildlife deer game hunting is economically important to the semi-arid South Texas region and is affected by climate change. In this paper, we model and investigate the effect of climate change on the spread of CWD using typical climate scenarios. We use a system of impulsive differential equations to depict the transmission of CWD between different age groups and gender of cervids. The carrying capacity and contact rates are assumed to depend on climate. Due to the polygamy of bucks, we use mating rates that depend on the number of bucks and does. We analyze the stability of the model and use simulations to study the effect of harvesting (culling) on eradicating the disease, given the climate of South Texas. We use typical climate change scenarios based on published data and our assumptions. For the climate indicator, we calculated and utilized the Standard Precipitation Evapotranspiration Index (SPEI). We found that climate change might hinder the efforts to reduce and effectively manage CWD as it becomes endemic to South Texas. The model shows the extinction of the deer population from this region is a likely outcome.

## Introduction

Chronic wasting disease (CWD) belongs to the family of transmissible spongiform encephalopathy (TSE) diseases, which also includes scrapie in sheep, bovine spongiform encephalopathy (BSE) in cattle, and variant Creutzfeldt-Jakob disease (vCJD) in humans (1). CWD is a fatal neurodegenerative disease affecting captive and free-ranging cervids in North America, with few cases reported in South Korea and three Nordic countries. CWD has been diagnosed in different species of cervids including: white-tailed deer (*Odocoileus virginianus*), black-tailed deer (*Odocoileus columbianus*), red deer (*Cervus elaphus*), mule deer (*Odocoileus hemionus*), Rocky Mountain elk (*Cervus elaphus nelsoni*), Shira's moose (*Alces alces Shirazi*), and Eurasian reindeer (*Rangifer tarandus tarandus*) (2).

In cervids, the infectious agent, PrP^CWD^, is found in the brain, spinal cord, neurons (3), skeletal muscle (4, 5), antler velvet (reported in elk) (6), and peripheral tissues (7). The main concern for CWD is the potential environmental transmission with the occurrence of infectious agents in the saliva, urine (8–10), blood (10), and feces (11, 12). Mule deer cumulatively shed as many prions in their feces as is found in their brain at the end of the incubation period (12). A secondary concern is that upon the animal's death, the carcass can act as a source of the infectious agent contributing to environmental contamination, creating “hotspots” for further transmission (10).

Symptoms of CWD are physical wasting, increased thirst and urination, excessive salivation, difficulty swallowing, trouble walking, drooping of ears, and changes in behavior (1). Experimentally, the CWD infectious agent can be readily transmitted directly between animals. Research with transgenic mice Tg(CerPrP) showed CWD infection with long incubation periods occurred through either nasal exposure (intranasal inoculation) (13) or oral inoculation by saliva or urine (8). Experts believe that saliva precedes feces in its efficiency of direct transmission, followed by urine and nasal discharge (14). Moreover, CWD horizontal transmission of disease can start in the pre-clinical stage (11, 15).

Environmental shedding of CWD prions is a route for transmission (16) that makes controlling and eradicating CWD difficult (14). CWD agent has been found in standing water in CWD endemic regions but at low titers (17). CWD agents can be shed into the environment through various routes including decaying cervid carcasses, cervid excreta, communal feeding stations, and shared bedding (14). Environmental contamination of CWD agents can remain in the soil for years (18, 19). PrP^CWD^ can adsorb in different types of soil components to varying degrees (20, 21). For example, prion binding to the soil mineral montmorillonite (Mte) greatly increases prion retention, bioavailability *in vivo*, and its infectivity (18, 22).

**Figure 1:**
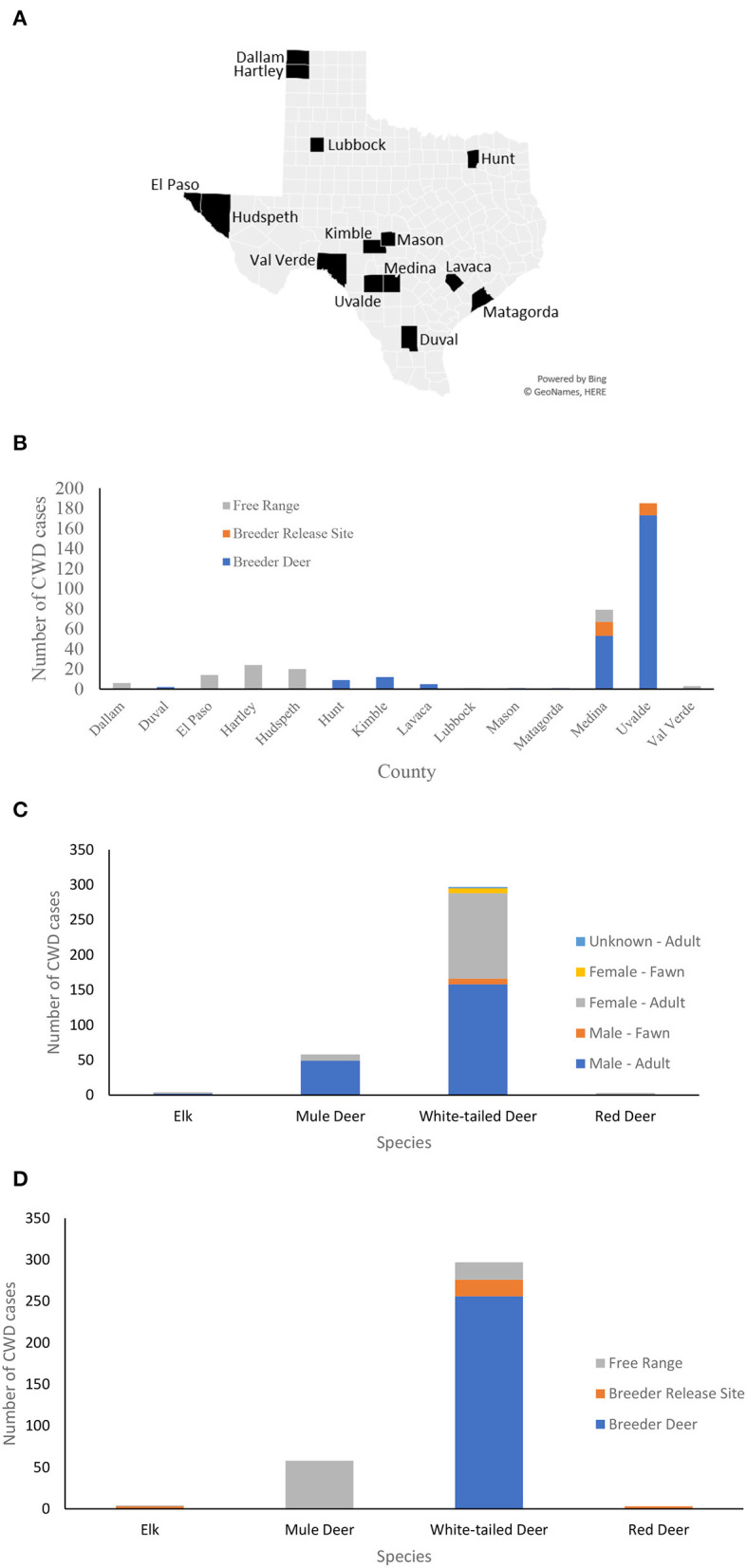
CWD cases in Texas. **(A)** A map of the counties in Texas where CWD was detected in captive and free-ranging deer. **(B)** Counts of CWD cases in each county by the site of the deer when diagnosed by CWD: breeder site, breeder release site, and in the free-range. **(C)** Counts of CWD cases by species, sex, and age group. The majority of CWD cases are adult white-tailed deer followed by mule deer. **(D)** Counts of CWD cases by species and site upon confirmed diagnosis.

Chronic wasting disease was first identified in 1967 at Fort Collins, Colorado, in a captive mule deer, since then it has been detected in 30 U.S. states and four Canadian provinces in free ranging cervids and commercial captive cervid facilities (23). The first CWD case in Hudspeth county, far West Texas was reported in 2012 in free-ranging mule deer and later in the adjacent county of El Paso, Texas, in 2017. As of March 2022, there have been 361 CWD cases reported in captive and free-ranging mule deer, red deer, white-tailed deer, and elk in Texas (24), see Figure 1.

The size of the South Texas area is about 37,800 square miles (60,833 square kilometers) and presents a challenging area for deer habitation. The semi-arid region's primary vegetation consists of thorny bushes such as mesquite, acacia, and prickly pear mixed with areas of grassland. The average annual rainfall is 20–32 inches (increasing from west to east) and the average annual net evaporation rate is 16–28 inches (25). The lack of water and foliage limits the carrying capacity of deer able to be supported.

The purpose of this research is to model the effects of climate change-induced temperature changes (with greater evaporation and more extreme weather events) to the spread of CWD to determine the long-term impacts on free-range deer populations in South Texas. The Texas Deer Association estimates the total impact of the deer industry on the Texas economy, including both breeding and hunting is $1.6 billion annually (26).

Modeling the spread of CWD in the wild has received a lot of attention. For example, work by Wasserberg et al. (27) studied the effectiveness of culling as a control measure. Moreover, Al-Arydah et al. (28, 29) studied gender-based harvesting, and Potapov et al. (30, 31) considered culling when different routes of transmissions were included along with genders of deer in case of frequency-dependent transmission and density-dependent transmission as well as density-dependent birth rates. In Oraby et al. (32) the authors used a model of seasonal culling with seasonal frequency-dependent and density-dependent transmissions, and impulsive birth to study CWD in Canada. Also, Vasilyeva et al. (33) included environmental routes of infections with clustering of the infectious agent in their CWD modeling. In recent study, Foley et al. (34) used a multi-stage deterministic matrix model to study the population size and disease prevalence in South Texas with its semi-arid environment over a 25-year.

The deer hunting season in South Texas occurs during the fall time—between the beginning of November and the third week of January (25). During the season, white-tailed deer breeding through polygamy takes place with a peak in late December. The gestation period is about seven and a half months followed by rearing for a few weeks till the fawn is able to walk—by the beginning of the fall. We consider a deer to be a fawn until 1 year of age at which point they are considered adults.

In this paper, we build and analyze a CWD disease spread model with sex and age group incorporated. It is a deterministic model to study the effect of CWD on the white-tailed deer population in South Texas. We use a system of ordinary differential equations and simulate different scenarios to understand how the disease can drive the population into extinction if not addressed. Our deterministic CWD transmission model considers climate-based carrying capacity and contact rates scenarios that can help to inform long-term deer population management. To maintain healthy free-range deer populations in South Texas, in the face of climate change and CWD, will require careful adaptive management practices.

## Model and Methods

### Model Description

**Figure 2:**
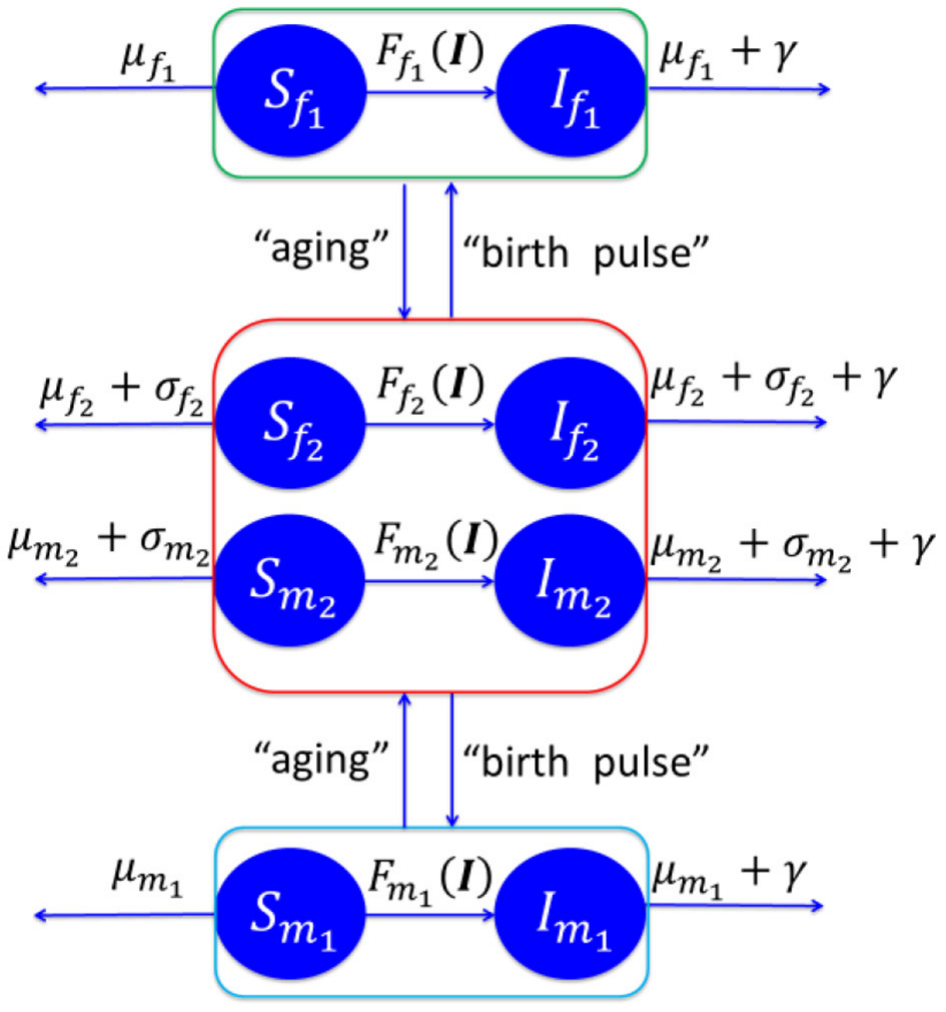
Schematic diagram of CWD disease spread in Texas. See Supplementary Material I for more details about the model and its analysis.

We postulate a susceptible-infectious (SI) model of the CWD spread dynamics between free-ranging deer, as shown in Figure 2. The model is based on eight compartments of susceptible female fawns *S*_*f*_1__, infectious female fawns *I*_*f*_1__, susceptible male fawns *S*_*m*_1__, infectious male fawns *I*_*m*_1__, susceptible female adult *S*_*f*_2__, infectious female adults *I*_*f*_2__, susceptible male adult *S*_*m*_2__, infectious male adult *I*_*m*_2__. We use the same notation for the number of deer in the eight compartments. The rates μ_*f*_1__, μ_*f*_2__, μ_*m*_1__, *and μ*_*m*_2__ are the natural death rates of the females (*f*) and males (*m*) classified by their age with two classes: fawns (1) and adults (2). Harvesting and hunting rates of adult deer, with the same classifications, are denoted by σ_*f*_2__, *and σ*_*m*_2__. The disease specific mortality rate is γ. The birth pulse rate is ν with female newborn fraction of *p*. See Supplementary Material I for more details about the model and its analysis.

We use a deterministic model comprised of a system of ordinary differential equations (ODE) with a birth pulse at time τ of each year. (We only consider the surviving newborns since those who do not make it through the first few weeks are not included in the process). Male deer are polygamous animals, which results in only a fraction of adult females being available for breeding if the number of adult male deer to the number of adult female deer exceeds the average number of male-to-female mating capacity, *c*.

Each susceptible deer in the subpopulation *i* (*i* = *f*_1_, *f*_2_, *m*_1_, and *m*_2_) endures a collective force of infection, including in the environment as fomites, perpetrated by the four infectious groups in which the total probability of infection is given by


$$\begin{array}{l} F_{i}\left(I\right)=\beta_{i}\left(\alpha_{i,f_{1}}\frac{I_{f_{1}}}{N_{f_{1}}}+\alpha_{{i,f}_{2}}\frac{I_{f_{2}}}{N_{f_{2}}}+\alpha_{{i,m}_{1}}\frac{I_{m_{1}}}{N_{m_{1}}}+\alpha_{i,m_{2}}\frac{I_{m_{2}}}{N_{m_{2}}}\right) \end{array}$$

which linearly combines their compartments' infectiousness with different degrees of contribution from the respective force. The degree of contribution is based on the contact rates $\alpha_{i,j}=\left(1-\lambda_{d}\right)\alpha_{i,j}^{\left(0\right)}+\lambda_{d}\alpha_{i,j}^{\left(1\right)}N_{j}$, for 0 ≤ λ_*d*_ ≤ 1. Contact rates are a linear combination of frequency and density dependent rates, $\alpha_{i,j}^{\left(0\right)}$ and $\alpha_{i,j}^{\left(1\right)}N_{j}$, respectively. We assume that at times of drought time with scarce foliage, deer will cluster at natural and human-made feeding locations. The coefficient $\alpha_{i,j}^{\left(1\right)}$ of contact's dependence on climate, may be due to feeding practices during drought, is modeled using $\alpha_{i,j}^{\left(1\right)}=\vartheta \;\alpha_{i,j}^{\left(0\right)}\frac{4-\Delta \left(n\right)}{8}\;.$ The variable Δ(*n*) is the Standardized Precipitation Evapotranspiration Index (SPEI) (−4 ≤ Δ(*n*) ≤ 4) in year *n*, see below. Dependence of $\alpha_{i,j}^{\left(1\right)}$ on SPEI might have a natural time lag but we leave that to empirical studies. The parameter ϑ is an adjustment to the transmission rate $\alpha_{i,j}^{\left(0\right)}$ for density-dependent modes of transmission.

There are, also, different probabilities of infections occurring denoted as β_*f*_1__, β_*f*_2__, β_*m*_1__, and β_*m*_2__ for each sex and age group. In the beginning of the epidemic, the environmental load is assumed to be proportional to the number of infectious and living deer, so we include the environmental route of transmission implicitly in the social transmission rates. We assume that any newborn of an infected doe becomes vertically infected at a rate of ρ.

We assume that harvesting and hunting of adult deer is seasonally taking place between the times τ_1_and τ_2_ of each year at rates $\sigma_{f_{2}}^{*}$ and $\sigma_{m_{2}}^{*}$. Natural death rates (μ_*f*_1__, μ_*f*_2__, μ_*m*_1__, *and μ*_*m*_2__), and disease specific mortality rate (γ) are also considered in the model. See Supplementary Material I, II for more details about the model, its parameters and analysis. See Supplementary Material III for model analysis in case of fixed climate inputs.

### Climate Data

Researchers have found a strong correlation between the Standard Precipitation Evapotranspiration Index (SPEI) as a measure of drought and different ecological elements like streamflow, soil moisture, forest growth, and crop yield (35). The SPEI almost always shows a value between −4 and 4. Negative values of the SPEI indicate times of drought whereas positive values indicate otherwise. We calculated the SPEI using the package called SPEI (https://cran.r-project.org/web/packages/SPEI/) in the statistical software R to find the annual mean SPEI following the method used by Hernandez and Uddameri (36). For hydrological data we used the available numbers from the Lawrence Livermore National Laboratory (http://gdo-dcp.ucllnl.org/downscaled_cmip3_projections) as a time series of the spatial mean for the South Texas region. The data is made from different weather variables, such as precipitation, maximum temperature, minimum temperature, potential evapotranspiration (PET), and other measures, available from the year 1950 and projected until 2099 in 97 scenarios. To find similar scenarios, we used a cluster analysis of SPEI's time series of 100 years (from 2000 to 2099) in conjunction with the K-means algorithm and the elbow method. We determined that K = 2 (Cluster 1 contains 54 scenarios and Cluster 2 contains 43 scenarios). To select a smaller number of representative scenarios from both clusters for further analysis we ran the Mann-Kendall test of monotonic trends and determined the most significant upward and downward trends in both clusters as well as the most non-significant (*p*−*value*>0.9) no-trend scenarios. We selected from Cluster 1: scenario 4 of τ = −0.72 and *p*−*value* = 5.4 × 10^−6^, with a downward trend. We selected from Cluster 2: scenario 74 of τ = −0.0016 and *p*−*value* = 0.92, with no trend; and scenario 97 of τ = 0.035 and *p*−*value* = 0.014, with an upward trend (See Supplementary Figure 1). We will also use a no-climate scenario for comparisons.

### Model Calibration

The model is calibrated in two steps. In the first step, a disease free, demographic version of the model is calibrated using the total annual number of cervids in South Texas. Data for cervid numbers were taken from the Texas Landowners Association (https://landassociation.org/how-many-deer-are-in-texas-white-tailed-deer-populations-listed-by-region/) for the time period of 2005–2017 under each one of the four climate scenarios including a no-climate-effect scenario (indicated as “no-climate” in the figure captions). Here, we use a measurement model to calibrate the following demographic parameters: ν, *K*, λ_*c*_, and μ_*i*_ (*i* = *f*_1_, *f*_2_, *m*_1_, and *m*_2_). We also calibrate the lag of time between the annual SPEI and birth rates. Finally, we calibrate the proportions of each age-sex group in the initial count of deer in 2005, assuming that proportion of male and female fawns are equal. The measurement model is that the actual annual number of cervid is normally distributed with mean given by the modeled total number of cervid and a heteroscedastic variance (ω*t*) that is proportional to the time. The choice of the heteroscedastic variance is based on the time series, the fitted trend, and the residuals after fitting the homoscedastic model. The parameter ϑ is calibrated so as to have the density-dependent mode of transmission's outcomes comparable to the frequency-dependent mode of transmission.

### Simulations and Results

We ran the model simulations under each of the four climate scenarios starting from 2005 to 2020 without a disease spread, as a burn-in period that initializes the population sizes in 2020. Then we started the disease spread simulations through the introduction of one infected male adult deer. We look at the disease and population evolution over time under the four climate scenarios with climate influence over the density dependent contact rate due to natural and human practices, like feeding sites. The first case is when the climate has no effect on contact rates. We also consider transmission rates with 2 and 5% of those rates provided in Supplementary Table 1.

**Figure 3:**
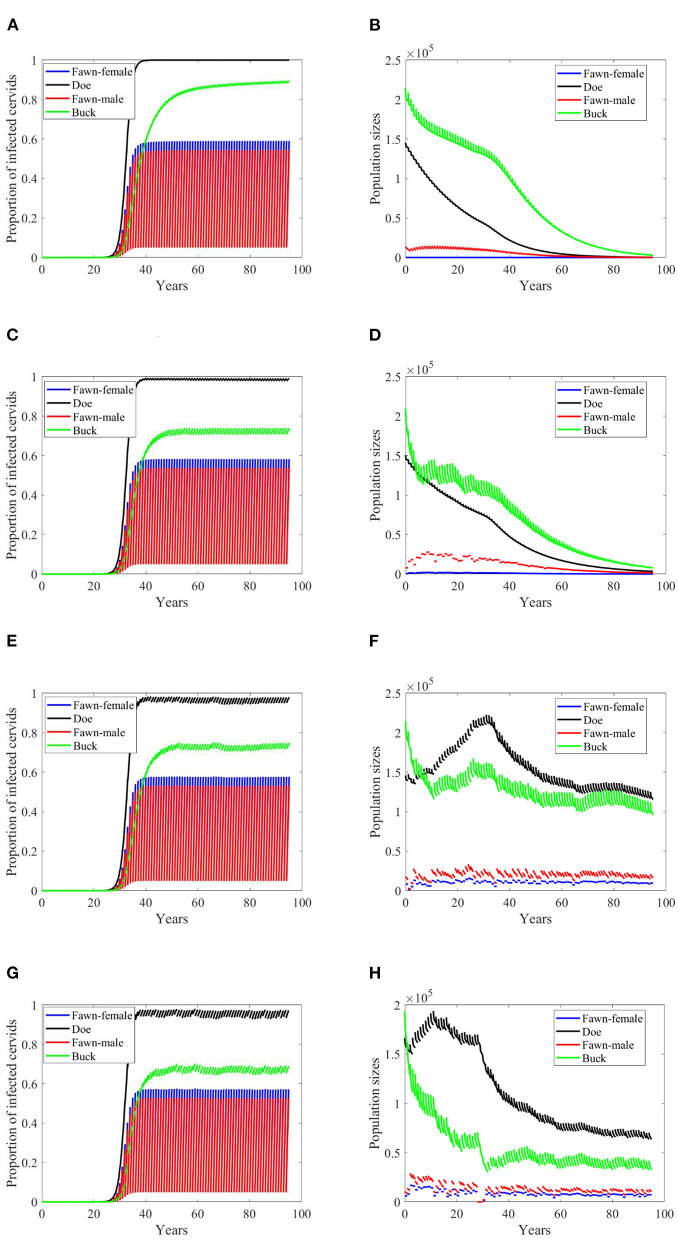
Number of infected cervids and population sizes classified by age-group and sex for different values for different climate scenarios. The simulations are for λ_*d*_ = 0 (only frequency-dependent transmission rates). The left panels show the number of infected cervids and the right panels show the population sizes. There are simulations for “no-climate” scenario **(A,B)**; scenario 4 in **(C,D)**; scenario 74 in **(E,F)**; and scenario 97 in **(G,H)**. The transmission rates are 5% of the ones in Supplementary Table 1. The rest of the parameters are given in Supplementary Table 1.

**Figure 4:**
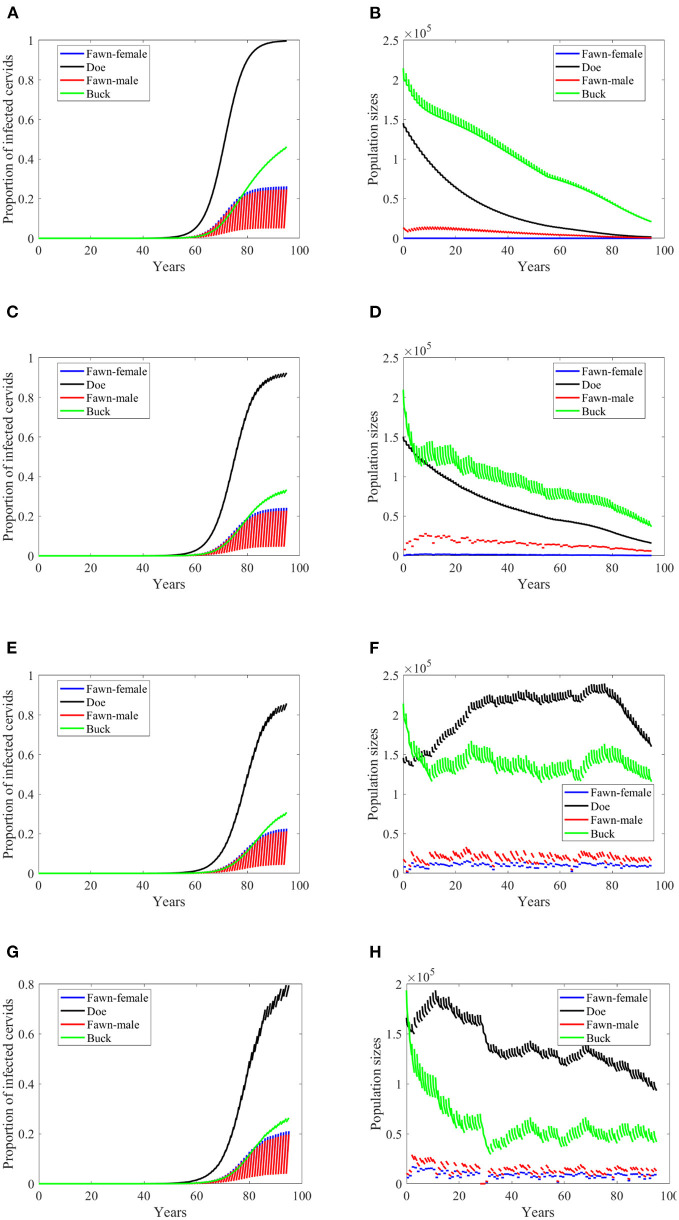
Number of infected cervids and population sizes classified by age-group and sex for different values for different climate scenarios. The simulations are for λ_*d*_ = 0 (only frequency-dependent transmission rates). The left panels show the number of infected cervids and the right panels show the population sizes. There are simulatons for “no-climate” scenario **(A,B)**; scenario 4 in **(C,D)**; scenario 74 in **(E,F)**; and scenario 97 in **(G,H)**. The transmission rates are 2% of the ones in Supplementary Table 1. The rest of the parameters are given in Supplementary Table 1.

**Figure 5:**
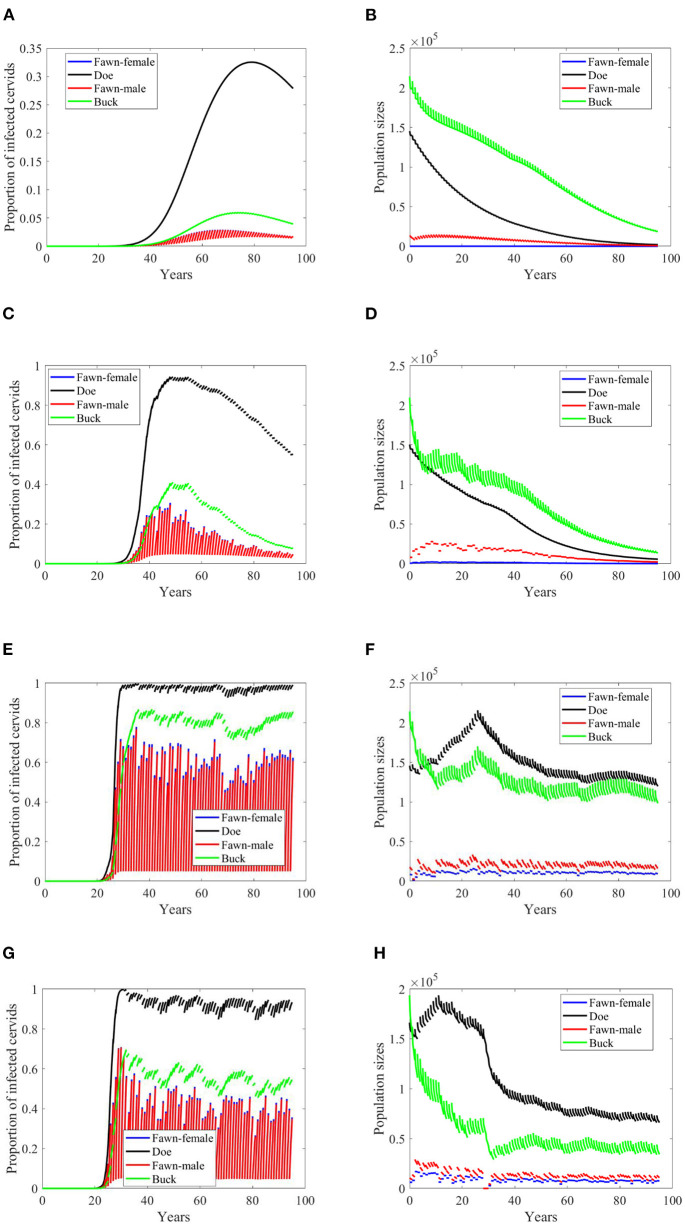
Number of infected cervids and population sizes classified by age-group and sex for different values for different climate scenarios. The simulations are for λ_*d*_ = 1 (only density-dependent transmission rates). The left panels show the number of infected cervids and the right panels show the population sizes. There are simulations for “no-climate” scenario **(A,B)**; scenario 4 in **(C,D)**; scenario 74 in **(E,F)**; and scenario 97 in **(G,H)**. The transmission rates are 5% of the ones in Supplementary Table 1. The rest of the parameters are given in Supplementary Table 1.

**Figure 6:**
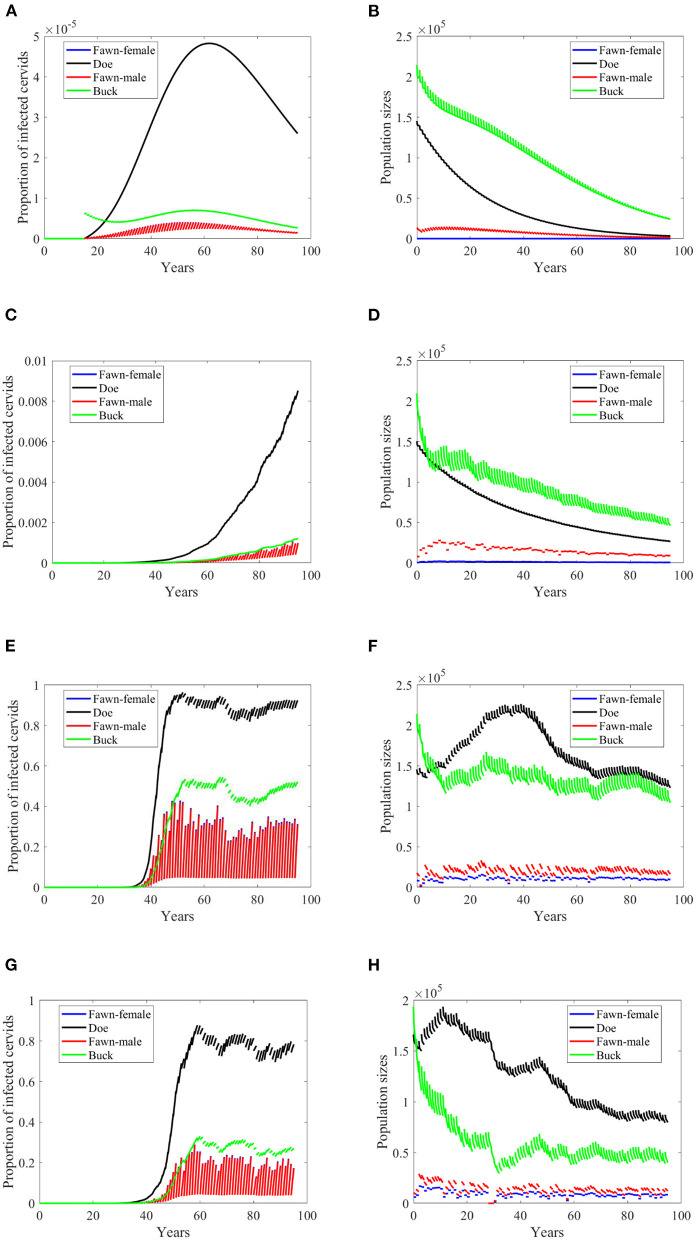
Number of infected cervids and population sizes classified by age-group and sex for different values for different climate scenarios. The simulations are for λ_*d*_ = 1 (only density-dependent transmission rates). The left panels show the number of infected cervids and the right panels show the population sizes. There are simulations for “no-climate” scenario **(A,B)**; scenario 4 in **(C,D)**; scenario 74 in **(E,F)**; and scenario 97 in **(G,H)**. The transmission rates are 2% of the ones in Supplementary Table 1. The rest of the parameters are given in Supplementary Table 1.

**Figure 7:**
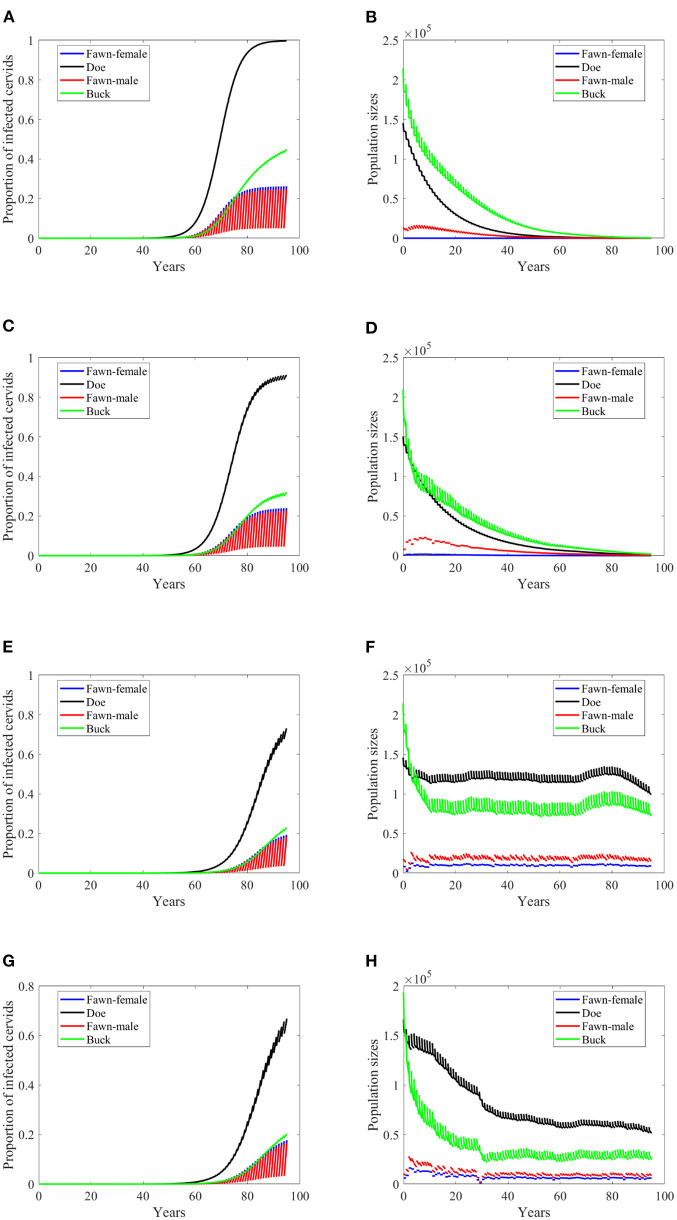
Number of infected cervids and population sizes classified by age-group and sex for different values for different climate scenarios. The simulations are for λ_*d*_ = 0 (only frequency-dependent transmission rates). The left panels show the number of infected cervids and the right panels are showing the population sizes. There are simulations for “no-climate” scenario **(A,B)**; scenario 4 in **(C,D)**; scenario 74 in **(E,F)**; and scenario 97 in **(G,H)**. The transmission rates are 2% of the ones in Supplementary Table 1, and harvesting rates increase by 100% above the ones in Supplementary Table 1. The rest of the parameters are given in Supplementary Table 1.

**Figure 8:**
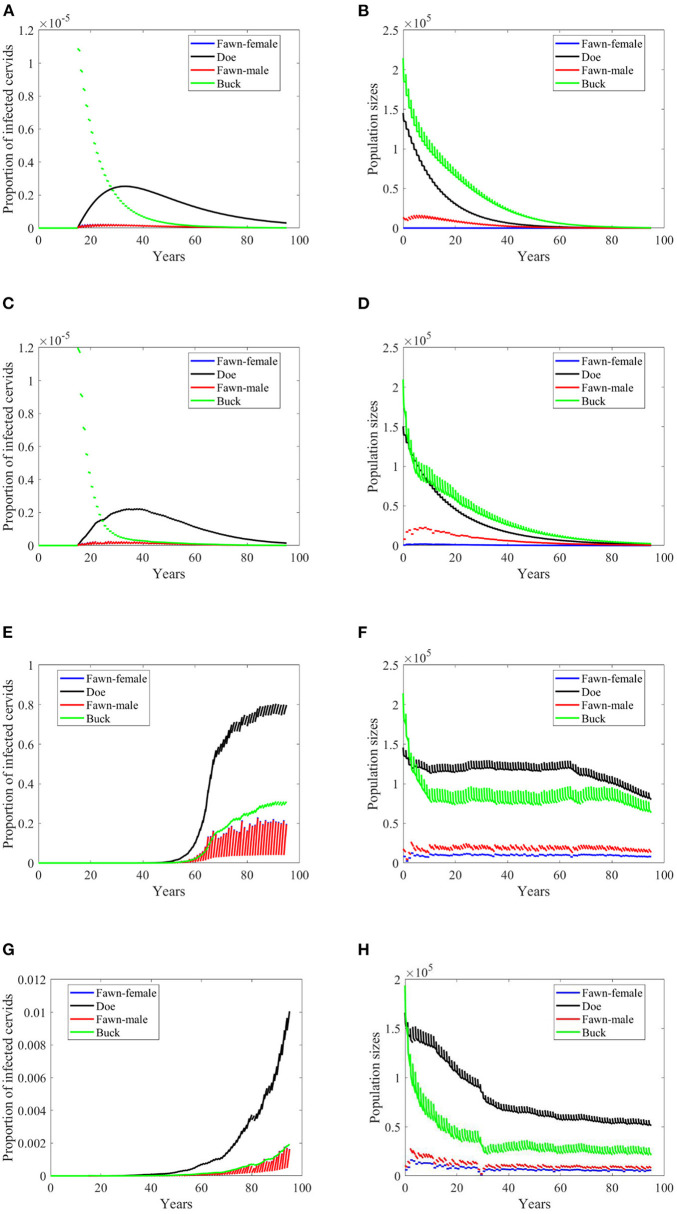
Number of infected cervids and population sizes classified by age-group and sex for different values for different climate scenarios. The simulations are for λ_*d*_ = 1 (only density-dependent transmission rates). The left panels show the number of infected cervids and the right panels show the population sizes. There are simulations for “no-climate” scenario **(A,B)**; scenario 4 in **(C,D)**; scenario 74 in **(E,F)**; and scenario 97 in **(G,H)**. The transmission rates are 2% of the ones in Supplementary Table 1, and harvesting rates increase by 100% above the ones in Supplementary Table 1. The rest of the parameters are given in Supplementary Table 1.

Based on Supplementary Table 1, while disease transmission rates, and death and harvesting rates are higher for male cervids (both for fawn and buck), seemingly the climate effect on the birth rates leads to a decaying population, Figures 3–**8B,D**. Disease prevalence is also taking stronger toll on female cervids due to their higher contact rates, Figures 3–8. Larger transmission rates result in lower population sizes, even for the Scenario 74 and 97 in which the is also highly prevalent, Figures 3, 4F,H.

While the prevalence of infected doe population sharply increases to a complete endemicity, in Figures 3E,G, the doe subpopulations do not go extinct but stabilize above but close to the size of the buck subpopulation, as shown in Figures 3F,H, after about 15 years since the first infected buck was introduced in 2020.

At a lower transmission rate, 2% of the transmission rates in Supplementary Table 1, the prevalence of infected cervid population slowed its rate of increase by about 5 times, Figures 4A,C,E,G. They also level up at a lower degree than those with 2.5 larger transmission rates, Figures 4A,C,E,G compared to Figures 3A,C,E,G. Prevalence of bucks is relatively slower and smaller. The total cervid population, however, does not seem to die out within a 100-year time period except on Figure 4B in which only the buck population survives longest with a deceasing population size. The skewed population sex ratio leads to decline in the birth and eventually the extinction of the whole population.

It can be also seen that while eventually the prevalence of infected doe subpopulation surpasses the prevalence in the buck subpopulation, the effect on the population is smaller during the 100-years, Figures 4F,H.

If the transmission mode is only density dependent, then the disease will not attain the same levels as it does in the first two scenarios, Figures 5A,C compared to Figures 3A,C, respectively. In Figure 5A, the prevalence does not only fail to reach those high levels as in Figure 3A, but also slowly climbs to the peak before it declines. The population, however, still dies out over time, Figure 5B. Which shows that the extinction is due to the carrying capacity's independence of the rain type of scenarios and the resulting calibrated parameters. Moreover, the prevalence increases at a slightly smaller rate in the no-climate scenario and in Scenario 4 in the density-dependent mode of transmission than in the frequency-dependent. The pattern turns the other way around for Scenarios 74 and 97. In the latter two scenarios, the prevalence shows oscillations due to weather, Figures 5E,G, but the population sizes do not show significant changes than those in frequency-dependent mode, Figures 5F,H.

Reduction in the transmission rate under the density-dependent transmission has mixed results in contrast to its effect under the frequency dependent mode. Under scenarios “no-climate,” 74 and 97, the reduction resulted in a peak or level reduction with a delay in Figures 6E,G, and hastening in Figure 6A. The populations in those three scenarios show slight increases in their sizes. Meanwhile, Scenario 4 showed a slow spread of the disease and significant difference in the population size. Comparatively, they are dissimilar to the case of frequency-dependent modes shown in Figures 3, 4.

Increasing harvesting rates is not a helpful measure of eradication in case of frequency dependent mode of transmission in general. That is true here when transmission rates are 5% of the ones in Supplementary Table 1 while doubling the harvesting rates (data are not shown since they are the same as the data shown in Figure 3). However, this idea fails to hold when transmission rates are 2% of the ones in Supplementary Table 1, due to the dependence of the carrying capacity on climate. That is, doubling the harvesting rate has similar effect on the prevalence as of lowering the transmission rates in frequency dependent, but with more effect on the population size, Figures 7A–H.

In case of density-dependent transmission, changes in climate scenarios will result in mixed outcomes of doubling harvesting rates. Scenarios, “no-climate” and 4, result in disease eradication, and population extinction, Figures 8A–D. Scenario 74 shows reduction in the level of endemicity and population sizes, Figures 8E,F. Scenario 97 shows a decreased rate of growth of the prevalence and reduction in the population sizes, Figures 8G,H.

**Figure 9:**
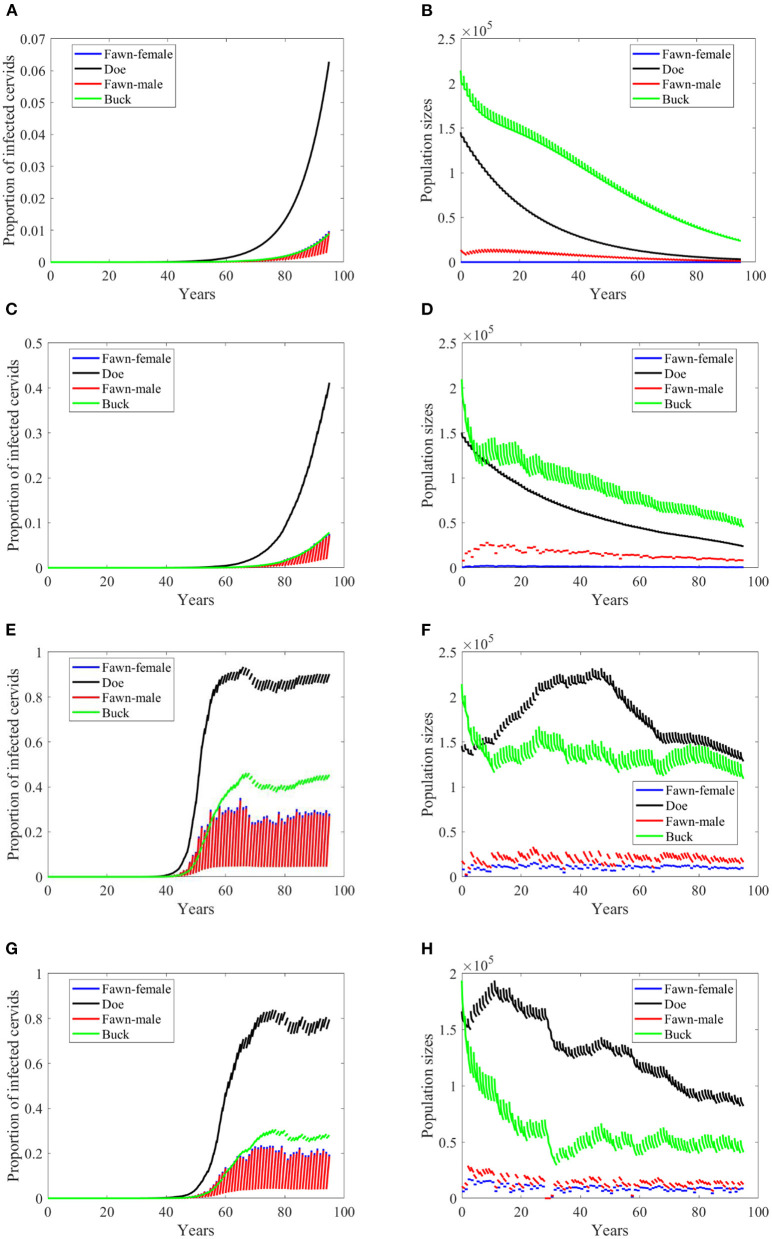
Number of infected cervids and population sizes classified by age-group and sex for different values for different climate scenarios. The simulations are for λ_*d*_ = 0.5. The left panels are showing the number of infected cervids and the right panels are showing the population sizes. There are simulations for “no-climate” scenario **(A,B)**; scenario 4 in **(C,D)**; scenario 74 in **(E,F)**; and scenario 97 in **(G,H)**. The transmission rates are 2% of the ones in Supplementary Table 1. The rest of the parameters are given in Supplementary Table 1.

When the transmission mode is a mixture of density dependent and frequency dependent, behavior of prevalence changes for some of the climate scenarios, as in Scenario “no-climate” and 4, Figures 9A,C. Prevalence in the other scenarios show further slower growth rates, Figures 9E,G. Populations sizes almost behave similarly, Figures 9B,D,F,H.

**Figure 10:**
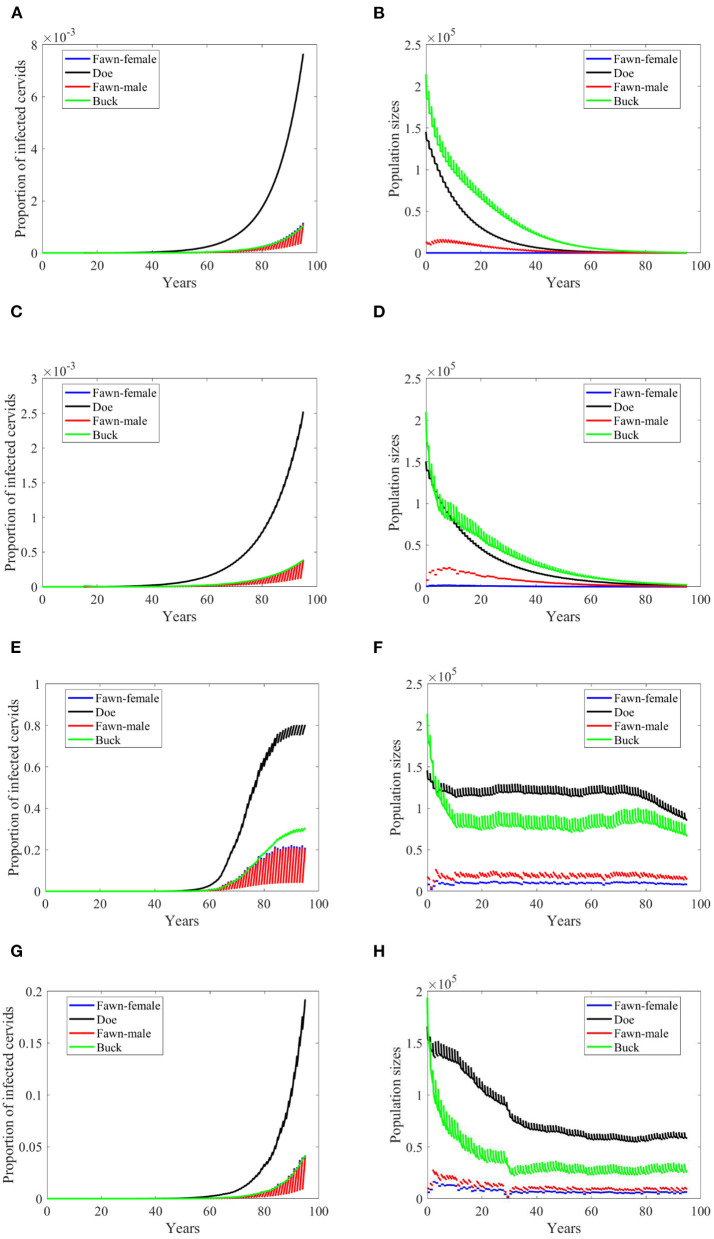
Number of infected cervids and population sizes classified by age-group and sex for different values for different climate scenarios. The simulations are for λ_*d*_ = 0.5. The left panels show the number of infected cervids and the right panels show the population sizes. There are simulations for “no-climate” scenario **(A,B)**; scenario 4 in **(C,D)**; scenario 74 in **(E,F)**; and scenario 97 in **(G,H)**. The transmission rates are 2% of the ones in Supplementary Table 1, and harvesting rates increase by 100% above the ones in Supplementary Table 1. The rest of the parameters are given in Supplementary Table 1.

Effects of doubling harvesting rates are closer to density dependent but with different effect under the climate scenarios “no-climate” and 4, Figures 10A,C. It looks that increasing harvesting rates can reduce the growth rate of the prevalence, Figures 10A,C,E,G. It can also reduce the level of endemicity as in Scenario 74, Figure 10E. Populations sizes in Figures 10B,D,F,H are comparable in shape to those in Figures 8B,D,F,H, but are slightly larger. Also, they do not show significant change to those in Figures 7B,D,F,H produced under frequency-dependent transmission rates, suggesting that increased culling will assist in decreasing prevalence under the different modes of transmission with little sensitivity in population dynamics.

## Discussion

We introduced a gender-based, multi-group compartmental model of CWD with impulsive birth and seasonal hunting parameters. The model used a limited mating strategy for bucks and incorporated the SPEI as a climate indicator. We selected different typical climate scenarios and used them to explore different climate effects on the course of the CWD epidemic as well as the cervid population in South Texas. Some of the model's parameters were calibrated under the selected climate scenarios. We depicted a carrying capacity that is climate dependent to investigate its effect on the disease and population sizes. We also introduced a climate-based contact rate in which unnatural feeding practices due to droughts and induced foliage scarcity can encourage a density dependent contact. Model simulation and analysis were performed to explore some of the anticipated patterns and how those patterns change with increasing harvesting during the hunting season.

Model simulations have shown mixed results for the different combinations of climate and harvesting scenarios and climatic influence over carrying capacity and contact rates. In these simulations, we found two dominant patterns: disease persistence with, and without population extinction. None of the simulations and parameter searches using Latin hypercube sampling and the stability results shown in Supplementary Material III indicated disease eradication and population survival. Additional harvesting can slow the epidemic and lower its persistence in many cases but can also drive the population to extinction during unfavorable conditions.

The model has its own strengths and limitations. Among the strengths is that the model uses climate indicators to examine the effect of harvesting on disease eradication and population extinction. Moreover, it acknowledges the effect of the fraction of the number of bucks to does on the reproduction of cervids. It also includes human feeding practices due to drought and its effect on the increase of contacts. Among the limitations is the need to have detailed cervid population data to estimate and parametrize the model. Also, the model might benefit from an explicit climate change model rather than using external scenarios, so as to examine the long-term courses of the population sizes and disease prevalence.

Argue et al. (37) noted that shared equipment, breeding herd, and forage in feeders were important risk factors for within farm transmission of CWD. Large free-range farms cultivate areas of foliage to attract deer for hunting and to support healthier free ranging animals. The time from the introduction of infected cervid until depopulation of the herd might also increase the risk of transmission. Hence, changing cervid-farming protocols might be a good control measure to reduce CWD spread on farms. Selective culling in free-ranging cervids, on the other hand, was shown to be effective in some cases since it reduces the spread of CWD in nature (38).

Different management strategies to reduce CWD have been tried in other U.S. states with varying levels of success. In Texas, the Texas Parks and Wildlife Department and the Texas Animal Health Commission have developed a management strategy for CWD in captive and free-ranging cervid populations. The management strategies are described as “evergreen” due to the ongoing management adjustments based on the animals' epidemiology.

The main goals of the CWD management plan are: effective management of CWD and other infectious disease through stakeholder engagement to minimize direct and indirect impacts of CWD to hunting, and to improve conservation efforts and economics related to hunting (25).

Strategic localized culling, in addition to traditional hunting, has been shown to stabilize CWD prevalence in some endemic areas. Comparative culling strategies between Illinois and Wisconsin white-tailed deer populations revealed localized culling is a disease management strategy that can maintain low disease prevalence while minimizing impacts on recreational deer harvest (39). Texas is adopting a management strategy of containment zones that limit unnatural movement of live CWD susceptible species and carcass parts to prevent spread. Cervid population management attempts to ensure strategies are at or below the habitat's carrying capacity with increased surveillance to monitor disease prevalence and location over time (25). The approach of adaptive management using containment zones will be increasingly difficult to implement going forward due to climate change impacts that will continuously and gradually reduce the habitat's carrying capacity over time.

Understanding how exactly climate affects carrying capacity and the spread of CWD in the semi-arid climate of South Texas is important to predict the success of culling strategies in controlling and delaying the disease spread, at least until other disease eradication methods become available (e.g., vaccine or gene therapy against CWD). Increased surveillance and monitoring programs (e.g., testing and reporting systems) will also be required to inform the emerging CWD wildlife epidemic in South Texas.

## Data Availability Statement

Publicly available datasets were analyzed in this study. This data can be found here: https://tpwd.texas.gov/huntwild/wild/diseases/cwd/tracking/; https://tpwd.texas.gov/regulations/outdoor-annual/hunting/.

## Author Contributions

MI, UB, MT, and TO contributed to conception and design of the study and analyzed the outcomes of the simulation. MI, UB, and TO established the model. MI and TO coded and analyzed the model and organized the data. TO performed the statistical analyses. All authors wrote the first draft of the manuscript, contributed to manuscript revision, read, and approved the submitted version.

## Conflict of Interest

The authors declare that the research was conducted in the absence of any commercial or financial relationships that could be construed as a potential conflict of interest.

## Publisher's Note

All claims expressed in this article are solely those of the authors and do not necessarily represent those of their affiliated organizations, or those of the publisher, the editors and the reviewers. Any product that may be evaluated in this article, or claim that may be made by its manufacturer, is not guaranteed or endorsed by the publisher.
