# Supplementary material for: Modeling the Impact of Climate Change on Cervid Chronic Wasting Disease in Semi-Arid South Texas
Source: Front Epidemiol. 2022 May 26;2:889280. doi: 10.3389/fepid.2022.889280 (PMC10910938; doi:10.3389/fepid.2022.889280)
Supplement: Supplementary file 1 [file Data_Sheet_1.pdf]

Modeling the Impact of Climate Change on Cervid Chronic Wasting Disease in Semi-Arid South Texas

Md Rafiul Islam[a,†], Ummugul Bulut[b,†], Teresa Patricia Feria-Arroyo[c], Michael G. Tyshenko[d], and Tamer Oraby[e]

[a] Department of Mathematics, Iowa State University, Ames, IA, USA

[b] Department of Mathematical, Physical, and Engineering Sciences, Texas A&M University-San Antonio, San Antonio, TX, USA

[c] Department of Biology, The University of Texas Rio Grande Valley, Edinburg, TX, USA

[d] Risk Sciences International, Ottawa, ON K1P 5J6 Canada

[e] School of Mathematical and Statistical Sciences, The University of Texas Rio Grande Valley, Edinburg, TX, USA

† These authors share first authorship

Supplementary Material I: Model Description

The dynamics of CWD spread among white-tailed deer is described by our birth-pulse susceptible-infectious (SI) model. The population is comprised of eight compartments of susceptible female fawns S_{f_1}, infectious female fawns with a number of I_{f_1}, susceptible male fawns with a number of S_{m_1}, infectious male fawns with a number of I_{m_1}, susceptible does (female adult deer) with a number of S_{f_2}, infectious does with a number of I_{f_2}, susceptible bucks (male adult deer) with a number of S_{m_2}, infectious bucks with a number of I_{m_2}. To model the disease and population dynamics, we use a system of ordinary differential equations. We also postulate a birth pulse at time τ of each year. That birth pulse happens with a rate of ν with saturation and a fraction p of all offspring are female. The birth rate is assumed to be dependent on the density of adult deer with a carrying capacity of $K(n)$ that depends linearly on annual climatic factors through the process $\Delta(\mathrm{n})$ in year n, which might be stochastic due to irregularity in climate. Vertical transmission and relative contribution of the different compartments

to infections are incorporated in the model. Natural death rates, harvesting/hunting rates, and CWD-specific mortality rate are also included, see Table S1 in Supplementary Material II.

$$\frac{dS_{f_1}}{dt} = -\beta_{f_1} S_{f_1}\left(\alpha_{f_1,f_1}\frac{I_{f_1}}{N_{f_1}} + \alpha_{f_1,f_2}\frac{I_{f_2}}{N_{f_2}} + \alpha_{f_1,m_1}\frac{I_{m_1}}{N_{m_1}} + \alpha_{f_1,m_2}\frac{I_{m_2}}{N_{m_2}}\right) - \mu_{f_1} S_{f_1} \quad \text{(S1.1)}$$

$$\frac{dI_{f_1}}{dt} = \beta_{f_1} S_{f_1}\left(\alpha_{f_1,f_1}\frac{I_{f_1}}{N_{f_1}} + \alpha_{f_1,f_2}\frac{I_{f_2}}{N_{f_2}} + \alpha_{f_1,m_1}\frac{I_{m_1}}{N_{m_1}} + \alpha_{f_1,m_2}\frac{I_{m_2}}{N_{m_2}}\right) - \left(\mu_{f_1} + \gamma\right) I_{f_1} \quad \text{(S1.2)}$$

$$\frac{dS_{f_2}}{dt} = -\beta_{f_2} S_{f_2}\left(\alpha_{f_2,f_1}\frac{I_{f_1}}{N_{f_1}} + \alpha_{f_2,f_2}\frac{I_{f_2}}{N_{f_2}} + \alpha_{f_2,m_1}\frac{I_{m_1}}{N_{m_1}} + \alpha_{f_2,m_2}\frac{I_{m_2}}{N_{m_2}}\right) - \left(\mu_{f_2} + \sigma_{f_2}\right) S_{f_2} \quad \text{(S1.3)}$$

$$\frac{dI_{f_2}}{dt} = \beta_{f_2} S_{f_2}\left(\alpha_{f_2,f_1}\frac{I_{f_1}}{N_{f_1}} + \alpha_{f_2,f_2}\frac{I_{f_2}}{N_{f_2}} + \alpha_{f_2,m_1}\frac{I_{m_1}}{N_{m_1}} + \alpha_{f_2,m_2}\frac{I_{m_2}}{N_{m_2}}\right) - \left(\mu_{f_2} + \sigma_{f_2} + \gamma\right) I_{f_2} \quad \text{(S1.4)}$$

$$\frac{dS_{m_1}}{dt} = -\beta_{m_1} S_{m_1}\left(\alpha_{m_1,f_1}\frac{I_{f_1}}{N_{f_1}} + \alpha_{m_1,f_2}\frac{I_{f_2}}{N_{f_2}} + \alpha_{m_1,m_1}\frac{I_{m_1}}{N_{m_1}} + \alpha_{m_1,m_2}\frac{I_{m_2}}{N_{m_2}}\right) - \mu_{m_1} S_{m_1} \quad \text{(S1.5)}$$

$$\frac{dI_{m_1}}{dt} = \beta_{m_1} S_{m_1}\left(\alpha_{m_1,f_1}\frac{I_{f_1}}{N_{f_1}} + \alpha_{m_1,f_2}\frac{I_{f_2}}{N_{f_2}} + \alpha_{m_1,m_1}\frac{I_{m_1}}{N_{m_1}} + \alpha_{m_1,m_2}\frac{I_{m_2}}{N_{m_2}}\right) - \left(\mu_{m_1} + \gamma\right) I_{m_1} \quad \text{(S1.6)}$$

$$\frac{dS_{m_2}}{dt} = -\beta_{m_2} S_{m_2}\left(\alpha_{m_2,f_1}\frac{I_{f_1}}{N_{f_1}} + \alpha_{m_2,f_2}\frac{I_{f_2}}{N_{f_2}} + \alpha_{m_2,m_1}\frac{I_{m_1}}{N_{m_1}} + \alpha_{m_2,m_2}\frac{I_{m_2}}{N_{m_2}}\right) - \left(\mu_{m_2} + \sigma_{m_2}\right) S_{m_2} \quad \text{(S1.7)}$$

$$\frac{dI_{m_2}}{dt} = \beta_{m_2} S_{m_2}\left(\alpha_{m_2,f_1}\frac{I_{f_1}}{N_{f_1}} + \alpha_{m_2,f_2}\frac{I_{f_2}}{N_{f_2}} + \alpha_{m_2,m_1}\frac{I_{m_1}}{N_{m_1}} + \alpha_{m_2,m_2}\frac{I_{m_2}}{N_{m_2}}\right) - \left(\mu_{m_2} + \sigma_{m_2} + \gamma\right) I_{m_2} \quad \text{(S1.8)}$$

$$B(n+\tau) = \nu\left(1 - \frac{1}{K + \lambda_c\,\Delta(n)}\left(N_{f_2}(n+\tau) + N_{m_2}(n+\tau)\right)\right) \quad \text{(S1.9)}$$

$$S_{f_1}((n+\tau)^+) = p\,B(n+\tau) \times$$

$$\left(S_{f_2}(n+\tau) + (1-\rho)\,I_{f_2}(n+\tau)\right)\frac{cN_{m_2}(n+\tau)}{1+cN_{m_2}(n+\tau)+N_{f_2}(n+\tau)} \qquad \text{(S1.10)}$$

$$I_{f_1}((n+\tau)^+) = p\,B(n+\tau) \times$$

$$\rho\,I_{f_2}(n+\tau)\,\frac{cN_{m_2}(n+\tau)}{1+cN_{m_2}(n+\tau)+N_{f_2}(n+\tau)} \qquad \text{(S1.11)}$$

$$S_{f_2}((n+\tau)^+) = S_{f_2}(n+\tau) + S_{f_1}(n+\tau) \qquad \text{(S1.12)}$$

$$I_{f_2}((n+\tau)^+) = I_{f_2}(n+\tau) + I_{f_1}(n+\tau) \qquad \text{(S1.13)}$$

$$S_{m_1}((n+\tau)^+) = (1-p)\,B(n+\tau) \times$$

$$\left(S_{f_2}(n+\tau) + (1-\rho)\,I_{f_2}(n+\tau)\right)\frac{cN_{m_2}(n+\tau)}{1+cN_{m_2}(n+\tau)+N_{f_2}(n+\tau)} \qquad \text{(S1.14)}$$

$$I_{m_1}((n+\tau)^+) = (1-p)B(n+\tau) \times$$

$$\rho\,I_{f_2}(n+\tau)\frac{cN_{m_2}(n+\tau)}{1+cN_{m_2}(n+\tau)+N_{f_2}(n+\tau)} \qquad \text{(S1.15)}$$

$$S_{m_2}((n+\tau)^+) = S_{m_2}(n+\tau) + S_{m_1}(n+\tau) \qquad \text{(S1.16)}$$

$$I_{m_2}((n+\tau)^+) = I_{m_2}(n+\tau) + I_{m_1}(n+\tau) \qquad \text{(S1.17)}$$

for $n < t \leq n+1$ and $n = 0, 1, 2, \ldots$. Also, the seasonal harvesting is given by

$$\sigma_{f_2}(t) = \begin{cases} \sigma_{f_2}^*, & \tau_1 < t \leq \tau_2 \\ 0, & otherwise. \end{cases} \qquad \text{(S1.18)}$$

$$\sigma_{m_2}(t) = \begin{cases} \sigma_{m_2}^*, & \tau_1 < t \leq \tau_2 \\ 0, & otherwise. \end{cases} \qquad \text{(S1.19)}$$

Where the total size of any of the four sub-populations $N_i(t) = S_i(t) + I_i(t)$ is the sum of the number of susceptible $S_i(t)$ and infected $I_i(t)$ deer in each sub-population i and i is

one of the sub-populations f_1, f_2, m_1, and m_2. Note that, $\alpha_{i,j} = (1 - \lambda_d)\alpha_{i,j}^{(0)} + \lambda_d \alpha_{i,j}^{(1)} N_j$, for $0 \leq \lambda_d \leq 1$.

We include horizontal (deer-to-deer except for progeny route) and vertical transmission (progeny route) of CWD as two modes of infection. Also, since in the beginning of the epidemic the environmental load will be proportional to the number of infectious and living deer, so in the short term, we include the environmental route of transmission implicitly in the social transmission rates.

Supplementary Material II: Data and Definition of Parameters and their Values

(A)

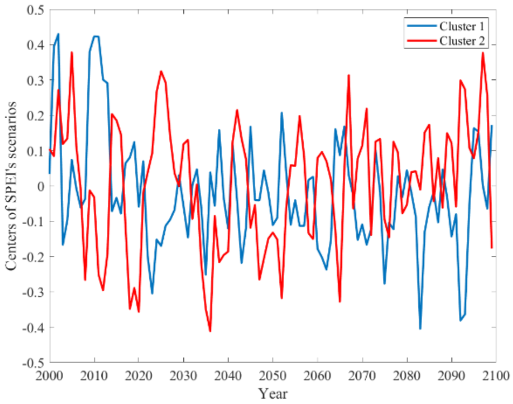

(B)

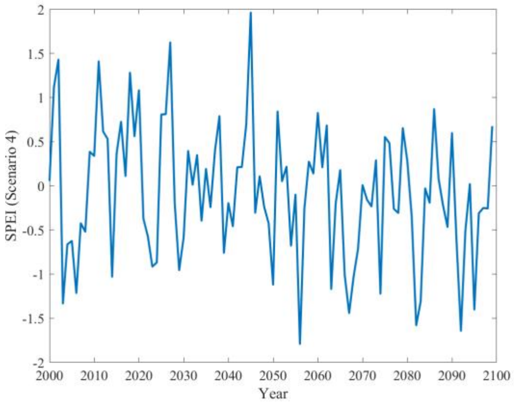

(C)

(D)

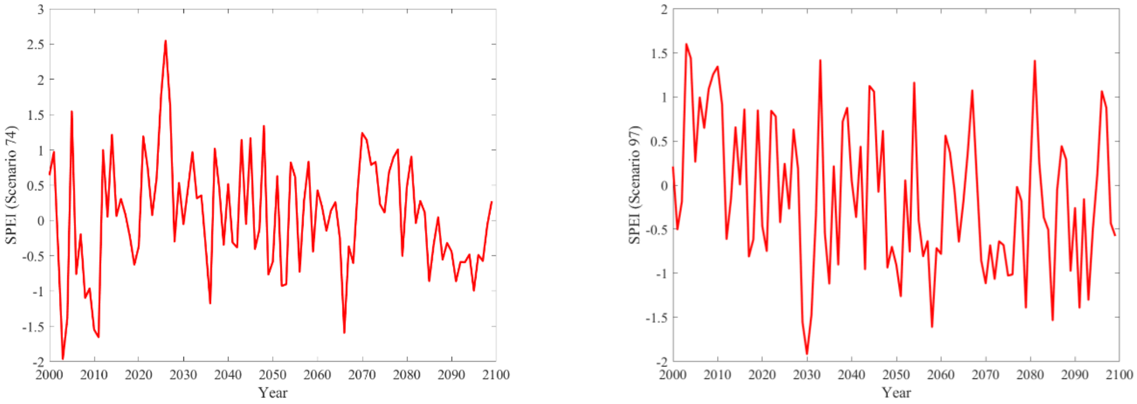

Figure S1. K-means cluster analysis of the 97 scenarios and Mann-Kendall tests of selected scenarios. Centers of Clusters 1 and 2 in (A). In Cluster 1: scenario 4 of $\tau = -.072$ and $p - value = 5.4 \times 10^{-6}$, with a downward trend (B). In Cluster 2: scenario 74 of $\tau = -.0016$ and $p - value = .92$, with no trend (C); and scenario 97 of $\tau = .035$ and $p - value = .014$, with an upward trend (D). In total five of the 97 scenarios were selected for comparison against the no-climate scenario for comparison.

The following is a table of the parameters, their role in the model with their values used in simulations and sensitivity analyses as well as their sources.

Table S1. Definition of parameters, their values, and sources. Time is given in month units.

	Parameters	Definition	Values	Units	Source
Demographic related	ν	Birth rate	Scenario dependent	fawn/doe/ pulse	Calibrated
	K	The carrying capacity or the degree density dependence in absence of climate effect	Scenario dependent	N/A	Calibrated
	λ_c	Degree of sensitivity of carrying capacity to climate change	Scenario dependent	N/A	Calibrated
	c	Average number of females breed by one male	2	N/A	assumed
	τ	Time of birth pulse	.5	N/A	
	p	Fraction of female newborn	.5	N/A	(Freddy *et al.*, 1993)
	μ_{f_1}	Natural death rate of female fawns	.0135	1/month	Calibrated
	μ_{f_2}	Natural death rate of doe	1/(12*15)	1/month	Calibrated

	μ_{m_1}	Natural death rates of male fawns	.0135	1/month	Calibrated
	μ_{m_2}	Natural death rate of buck	1/(12*11)	1/month	Calibrated
	$\sigma_{f_2}^*$	Harvesting rates of does	.16	1/month	(Williams *et al.*, 2002)
	$\sigma_{m_2}^*$	Harvesting rates of buck	.32	1/month	(Williams *et al.*, 2002)
	τ_1	Beginning time of harvesting season	0 – Nov 6	N/A	(TPDW, 2021)
	τ_2	End time of harvesting season	71/365 – Jan 16	N/A	(TPDW, 2021)
	$\alpha^{(0)} = (\alpha_{i,j}^{(0)})$	Matrix of contact rates[1] between and within f_1, f_2, m_1, and m_2	$\begin{pmatrix} 50 & 36 & 40 & 3 \\ 36 & 48 & 30 & 5 \\ 40 & 30 & 14 & 2 \\ 3 & 5 & 2 & 4 \end{pmatrix}$	1/month	(Belsare and Stewart, 2020)
	ϑ	Adjustment parameter of contact rates to density dependence	0.000026	N/A	Calibrated
	λ_d	Degree of sensitivity of density-dependent contact rate to climate change	0-1	N/A	Assumed
**Disease related**	β_{f_1}	Transmission probability to female fawns	0.255*	N/A	(Jennelle *et al.*, 2014)
	β_{f_2}	Transmission probability to does	0.255*	N/A	(Jennelle *et al.*, 2014)
	β_{m_1}	Transmission probability to male fawns	0.305*	N/A	(Jennelle *et al.*, 2014)
	β_{m_2}	Transmission probability to bucks	0.305*		(Jennelle *et al.*, 2014)
	ρ	Probability of vertical transmission	0.05	NA	(Miller *et al.*,, 2000)
	γ	CWD-specific mortality rate	1/24= 0.04166	1/montsh	(Foley *et al.*, 2016; Potapov *et al.*, 2016)

[1] Through social and environmental modes.

Supplementary Material III: Model Analysis

Let us assume that the year starts on the day before the birth pulse. Since the total size of any of the four sub-populations $N_i(t) = S_i(t) + I_i(t)$, we have

$$\frac{dN_i}{dt} = \frac{dI_i}{dt} + \frac{dS_i}{dt}$$

and the rate of change of the disease prevalence $P_i = \frac{I_i}{N_i}$ of each sub-population is thus given by

$$\frac{dP_i}{dt} = \frac{dI_i/dt}{N_i}\frac{S_i}{N_i} - \frac{dS_i/dt}{N_i}\frac{I_i}{N_i}$$

See (Roberts and Kao, 1998), and (Oraby et al., 2014). That leads to an equivalent system of equations to (S1.1-S1.16), with $\alpha_{i,j} = (1-\lambda_d)\alpha_{i,j}^{(0)} + \lambda_d \alpha_{i,j}^{(1)} N_j$, for $0 \leq \lambda_d \leq 1$, given by

$$\frac{dN_{f_1}}{dt} = -(\mu_{f_1} + \gamma P_{f_1})N_{f_1} \tag{S3.1}$$

$$\frac{dP_{f_1}}{dt} = (1-P_{f_1})\left[\beta_{f_1}(\alpha_{f_1,f_1}P_{f_1} + \alpha_{f_1,f_2}P_{f_2} + \alpha_{f_1,m_1}P_{m_1} + \alpha_{f_1,m_2}P_{m_2}) - \gamma P_{f_1}\right] \tag{S3.2}$$

$$\frac{dN_{f_2}}{dt} = -(\mu_{f_2} + \sigma_{f_2} + \gamma P_{f_2})N_{f_2} \tag{S3.3}$$

$$\frac{dP_{f_2}}{dt} = (1-P_{f_2})\left[\beta_{f_2}(\alpha_{f_2,f_1}P_{f_1} + \alpha_{f_2,f_2}P_{f_2} + \alpha_{f_2,m_1}P_{m_1} + \alpha_{f_2,m_2}P_{m_2}) - \gamma P_{f_2}\right] \tag{S3.4}$$

$$\frac{dN_{m_1}}{dt} = -(\mu_{m_1} + \gamma P_{m_1})N_{m_1} \tag{S3.5}$$

$$\frac{dP_{m_1}}{dt} = (1-P_{m_1})\left[\beta_{m_1}(\alpha_{m_1,f_1}P_{f_1} + \alpha_{m_1,f_2}P_{f_2} + \alpha_{m_1,m_1}P_{m_1} + \alpha_{m_1,m_2}P_{m_2}) - \gamma P_{m_1}\right] \tag{S3.6}$$

$$\frac{dN_{m_2}}{dt} = -(\mu_{m_2} + \sigma_{m_2} + \gamma P_{m_2})N_{m_2} \tag{S3.7}$$

$$\frac{dP_{m_2}}{dt} = \left(1 - P_{m_2}\right)\left[\beta_{m_2}\left(\alpha_{m_2,f_1}P_{f_1} + \alpha_{m_2,f_2}P_{f_2} + \alpha_{m_2,m_1}P_{m_1} + \alpha_{m_2,m_2}P_{m_2}\right) - \gamma P_{m_2}\right] \tag{S3.8}$$

for $t \geq 0$, $t \neq n$ and $n = 0, 1, 2, \ldots$

$$B(n) = \nu\left(1 - \frac{1}{K + \lambda_c\,\Delta(n)}\left(N_{f_2}(n) + N_{m_2}(n)\right)\right) \tag{S3.9}$$

$$N_{f_1}(n^+) = p\,N_{f_2}(n)B(n)\,\frac{cN_{m_2}(n)}{1 + cN_{m_2}(n) + N_{f_2}(n)} \tag{S3.10}$$

$$P_{f_1}(n^+) = \rho\,P_{f_2}(n) \tag{S3.11}$$

$$N_{f_2}(n^+) = N_{f_2}(n) + N_{f_1}(n) \tag{S3.12}$$

$$P_{f_2}(n^+) = \frac{N_{f_2}(n)\,P_{f_2}(n) + N_{f_1}(n)\,P_{f_1}(n)}{N_{f_2}(n) + N_{f_1}(n)} \tag{S3.13}$$

$$N_{m_1}(n^+) = (1 - p)\ \mathrm{B(n)}\ N_{f_2}(n)\frac{cN_{m_2}(n)}{1 + cN_{m_2}(n) + N_{f_2}(n)} \tag{S3.14}$$

$$P_{m_1}(n^+) = \rho\,P_{f_2}(n) \tag{S3.15}$$

$$N_{m_2}(n^+) = N_{m_2}(n) + N_{m_1}(n) \tag{S3.16}$$

$$P_{m_2}(n^+) = \frac{N_{m_2}(n)\,P_{m_2}(n) + N_{m_1}(n)\,P_{m_1}(n)}{N_{m_2}(n) + N_{m_1}(n)} \tag{S3.17}$$

We will study the local stability of equilibria when $\Delta(n)$ is constant. Let $X(t) = \left(N_{f_1}(t), P_{f_1}(t), N_{f_2}(t), P_{f_2}(t), N_{m_1}(t), P_{m_1}(t), N_{m_2}(t), P_{m_2}(t)\right)^T$ be the vector of the status of the population and epidemic at time t. Let $\bar{X}(t) = \left(\bar{N}_{f_1}(t), \bar{P}_{f_1}(t), \bar{N}_{f_2}(t), \bar{P}_{f_2}(t), \bar{N}_{m_1}(t), \bar{P}_{m_1}(t), \bar{N}_{m_2}(t), \bar{P}_{m_2}(t)\right)^T$ be a periodic solution vector of the system (S3.1-S3.8) which by perturbation with a vector $x(t)$ such that

$$x(t) = \mathbf{\Phi}(t)x(0) \tag{S3.18}$$

such that $\mathbf{\Phi}(0) = \mathbf{I}$, where $\mathbf{I}$ is the 8×8 identity matrix and $\mathbf{\Phi}$ is the fundamental matrix. That leads to the matrix-system of differential equations

$$\frac{d\mathbf{\Phi}(t)}{dt} = \mathbf{J}\big(\bar{X}(t)\big) \cdot \mathbf{\Phi}(t) \tag{S3.19}$$

such that $\mathbf{\Phi}(0) = \mathbf{I}$, where $\mathbf{J}(\bar{X})$ is the Jacobian of the system (S3.1-S3.8) evaluated at $\bar{X}$. The Jacobian matrix $\mathbf{J}(\bar{X})$ is given by

$$\begin{bmatrix} -(\mu_{f_1} + \gamma\bar{P}_{f_1}) & -\gamma\bar{N}_{f_1} & 0 & 0 & 0 & 0 & 0 & 0 \\ \vartheta_{f_1,f_1} & \Theta_{f_1} & \vartheta_{f_1,f_2} & \beta_{f_1}\alpha_{f_1,f_2}(1-\bar{P}_{f_1}) & \vartheta_{f_1,m_1} & \beta_{f_1}\alpha_{f_1,m_1}(1-\bar{P}_{f_1}) & \vartheta_{f_1,m_2} & \beta_{f_1}\alpha_{f_1,m_2}(1-\bar{P}_{f_1}) \\ 0 & 0 & -(\mu_{f_2} + \sigma_{f_2} + \gamma\bar{P}_{f_2}) & -\gamma\bar{N}_{f_2} & 0 & 0 & 0 & 0 \\ \vartheta_{f_2,f_1} & \beta_{f_2}\alpha_{f_2,f_1}(1-\bar{P}_{f_2}) & \vartheta_{f_2,f_2} & \Theta_{f_2} & \vartheta_{f_2,m_1} & \beta_{f_2}\alpha_{f_2,m_1}(1-\bar{P}_{f_2}) & \vartheta_{f_2,m_2} & \beta_{f_2}\alpha_{f_2,m_2}(1-\bar{P}_{f_2}) \\ 0 & 0 & 0 & 0 & -(\mu_{m_1} + \gamma\bar{P}_{m_1}) & -\gamma\bar{N}_{m_1} & 0 & 0 \\ \vartheta_{\mathrm{m}_1,f_1} & \beta_{m_1}\alpha_{m_1,f_1}(1-\bar{P}_{m_1}) & \vartheta_{\mathrm{m}_1,f_2} & \beta_{m_1}\alpha_{m_1,f_2}(1-\bar{P}_{m_1}) & \vartheta_{m_1,m_1} & \Theta_{m_1} & \vartheta_{m_1,m_2} & \beta_{m_1}\alpha_{m_1,m_2}(1-\bar{P}_{m_1}) \\ 0 & 0 & 0 & 0 & 0 & 0 & -(\mu_{m_2} + \sigma_{m_2} + \gamma\bar{P}_{m_2}) & -\gamma\bar{N}_{m_2} \\ \vartheta_{m_2,f_1} & \beta_{m_2}\alpha_{m_2,f_1}(1-\bar{P}_{m_2}) & \vartheta_{m_2,f_2} & \beta_{m_2}\alpha_{m_2,f_2}(1-\bar{P}_{m_2}) & \vartheta_{m_2,m_1} & \beta_{m_2}\alpha_{m_2,m_1}(1-\bar{P}_{m_2}) & \vartheta_{m_2,m_2} & \Theta_{m_2} \end{bmatrix}$$

where

$$\Theta_{f_1} = (\beta_{f_1}\alpha_{f_1,f_1} - \gamma)\big(1 - 2\,\bar{P}_{f_1}\big) - \beta_{f_1}\big(\alpha_{f_1,f_2}\bar{P}_{f_2} + \alpha_{f_1,m_1}\bar{P}_{m_1} + \alpha_{f_1,m_2}\bar{P}_{m_2}\big),$$

$$\Theta_{f_2} = (\beta_{f_2}\alpha_{f_2,f_2} - \gamma)\big(1 - 2\,\bar{P}_{f_2}\big) - \beta_{f_2}\big(\alpha_{f_2,f_1}\bar{P}_{f_1} + \alpha_{f_2,m_1}\bar{P}_{m_1} + \alpha_{f_2,m_2}\bar{P}_{m_2}\big),$$

$$\Theta_{m_1} = (\beta_{m_1}\alpha_{m_1,m_1} - \gamma)\big(1 - 2\,\bar{P}_{m_1}\big) - \beta_{m_1}\big(\alpha_{m_1,f_1}\bar{P}_{f_1} + \alpha_{m_1,f_2}\bar{P}_{f_2} + \alpha_{m_1,m_2}\bar{P}_{m_2}\big),$$

and

$$\Theta_{m_2} = (\beta_{m_2}\alpha_{m_2,m_2} - \gamma)\big(1 - 2\,\bar{P}_{m_2}\big) - \beta_{m_2}\big(\alpha_{m_2,f_1}\bar{P}_{f_1} + \alpha_{m_2,f_2}\bar{P}_{f_2} + \alpha_{m_2,m_1}\bar{P}_{m_1}\big).$$

Also,

$$\vartheta_{i,j} = (1 - \bar{P}_i)\,\beta_i\lambda_d\alpha_{i,j}^{(1)}\bar{P}_j$$

for all $i, j = f_1, f_2, m_1$, and m_2.

The monodromy matrix is given by $\boldsymbol{M} \coloneqq \boldsymbol{D}\big(\bar{X}(1)\big) \cdot \mathbf{\Phi}(1)$. The matrix $\boldsymbol{D}$ is the Jacobian matrix of the pulsive equations evaluated at the periodic solutions. It is given by

$$\begin{bmatrix} 0 & 0 & p\,\nu\,\Psi_1 & 0 & 0 & 0 & p\,\nu\,\Psi_2 & 0 \\ 0 & 0 & 0 & \rho & 0 & 0 & 0 & 0 \\ 1 & 0 & 1 & 0 & 0 & 0 & 0 & 0 \\ \bar{N}_{f_2}\frac{\bar{P}_{f_1} - \bar{P}_{f_2}}{(\bar{N}_{f_1} + \bar{N}_{f_2})^2} & \frac{\bar{N}_{f_1}}{\bar{N}_{f_1} + \bar{N}_{f_2}} & \bar{N}_{f_1}\frac{\bar{P}_{f_2} - \bar{P}_{f_1}}{(\bar{N}_{f_1} + \bar{N}_{f_2})^2} & \frac{\bar{N}_{f_2}}{\bar{N}_{f_1} + \bar{N}_{f_2}} & 0 & 0 & 0 & 0 \\ 0 & 0 & (1-p)\,\nu\,\Psi_1 & 0 & 0 & 0 & (1-p)\,\nu\,\Psi_2 & 0 \\ 0 & 0 & 0 & \rho & 0 & 0 & 0 & 0 \\ 0 & 0 & 0 & 0 & 1 & 0 & 1 & 0 \\ 0 & 0 & 0 & 0 & \bar{N}_{m_2}\frac{\bar{P}_{m_1} - \bar{P}_{m_2}}{(\bar{N}_{m_1} + \bar{N}_{m_2})^2} & \frac{\bar{N}_{m_1}}{\bar{N}_{m_1} + \bar{N}_{m_2}} & \bar{N}_{m_1}\frac{\bar{P}_{m_2} - \bar{P}_{m_1}}{(\bar{N}_{m_1} + \bar{N}_{m_2})^2} & \frac{\bar{N}_{m_2}}{\bar{N}_{m_1} + \bar{N}_{m_2}} \end{bmatrix}$$

Where

$$\Psi_1 = \left(1 - \frac{1}{\bar{K}}\big(\bar{N}_{f_2} + \bar{N}_{m_2}\big)\right)\frac{c\,\bar{N}_{m_2}\big(1 + c\,\bar{N}_{m_2}\big)}{\big(1 + c\bar{N}_{m_2} + \bar{N}_{f_2}\big)^2} - \frac{1}{\bar{K}}\bar{N}_{f_2}\,\frac{c\bar{N}_{m_2}}{1 + c\bar{N}_{m_2} + \bar{N}_{f_2}}$$

and

$$\Psi_2 = \left(1 - \frac{1}{\overline{K}}\left(\overline{N}_{f_2} + \overline{N}_{m_2}\right)\right)\frac{c\,\overline{N}_{f_2}\left(1+\overline{N}_{f_2}\right)}{\left(1+c\overline{N}_{m_2}+\overline{N}_{f_2}\right)^2} - \frac{1}{\overline{K}}\overline{N}_{f_2}\,\frac{c\overline{N}_{m_2}}{1+c\overline{N}_{m_2}+\overline{N}_{f_2}}$$

An equilibrium one-period solution $\bar{X}$ is locally stable if the eigenvalues of the monodromy matrix $\boldsymbol{M}$ have absolute values less than one.

Let

$$\bar{\sigma}_{f_2}(t) := \int_0^t \sigma_{f_2}(s)ds = \begin{cases} 0, & 0 < t < \tau_1 \\ \sigma_{f_2}^*(t-\tau_1), & \tau_1 \le t \le \tau_2 \\ \sigma_{f_2}^*(\tau_2-\tau_1), & \tau_2 \le t \le 1 \end{cases}$$

and

$$\bar{\sigma}_{m_2}(t) := \int_0^t \sigma_{m_2}(s)ds = \begin{cases} 0, & 0 < t < \tau_1 \\ \sigma_{m_2}^*(t-\tau_1), & \tau_1 \le t \le \tau_2 \\ \sigma_{m_2}^*(\tau_2-\tau_1), & \tau_2 \le t \le 1. \end{cases}$$

1) First, we consider the disease free periodic DFPE solution which is the only optimal solution for the disease spread problem. The DFPE is given by

$$\bar{X}_{DFPE}(t) = \left(\overline{N}_{f_1}(t), 0, \overline{N}_{f_2}(t), 0, \overline{N}_{m_1}(t), 0, \overline{N}_{m_2}(t), 0\right)^T,$$

where

$$\overline{N}_{f_1}(t) = \overline{N}_{f_1}(1^-)\, exp(-\mu_{f_1}(t-1)),$$

$$\overline{N}_{f_2}(t) = \overline{N}_{f_2}(1^-)\, exp\left(-\left(\mu_{f_2}(t-1) + \left(\bar{\sigma}_{f_2}(t) - \bar{\sigma}_{f_2}(1)\right)\right)\right),$$

$$\overline{N}_{m_1}(t) = \overline{N}_{m_1}(1^-) exp(-\mu_{m_1}(t-1)),$$

and

$$\overline{N}_{m_2}(t) = \overline{N}_{m_2}(1^-)\, exp\left(-\left(\mu_{m_2}(t-1) + \left(\bar{\sigma}_{m_2}(t) - \bar{\sigma}_{m_2}(1)\right)\right)\right).$$

Where,

$$\overline{N}_{f_1}(1^-) = \left(exp\left(\mu_{f_2} + \bar{\sigma}_{f_2}(1)\right) - 1\right) \overline{N}_{f_2}(1^-),$$

$$\overline{N}_{m_1}(1^-) = \left(exp\left(\mu_{m_2} + \bar{\sigma}_{m_2}(1)\right) - 1\right) \overline{N}_{m_2}(1^-),$$

$$\overline{N}_{m_2}(1^-) = Q\,\overline{N}_{f_2}(1^-).$$

Where $Q = \frac{1-p}{p}\frac{exp(\mu_{f_1})\left(exp\left(\mu_{f_2}+\bar{\sigma}_{f_2}(1)\right)-1\right)}{exp(\mu_{m_1})\left(exp\left(\mu_{m_2}+\bar{\sigma}_{m_2}(1)\right)-1\right)}$. The terminal size of adult female $\overline{N}_{f_2}(1^-)$ is the $(+)$ branch of the root of the quadratic equation

$$\frac{1}{\overline{K}}pcvQ(1+Q)x^2 - (pcvQ - cMQ - M)x + M = 0,$$

where

$$M = exp(\mu_{f_1})\left(exp\left(\mu_{f_2} + \bar{\sigma}_{f_2}(1)\right) - 1\right).$$

The only existence condition of the DFPE is easily found to be that the discriminant is non-negative; which is equivalent to

$$(cpQ)^2\nu^2 - 2cpQM\left[1 + \frac{2}{\overline{K}} + \left(c + \frac{2}{\overline{K}}\right)Q\right]\nu + M^2(1+cQ)^2 > 0 \qquad \text{(EC)}$$

That would result in

$$\mathbf{J}(\bar{X}_{DFPE}) = \begin{bmatrix} -\mu_{f_1} & -\gamma\overline{N}_{f_1} & 0 & 0 & 0 & 0 & 0 & 0 \\ 0 & \beta_{f_1}\bar{\alpha}_{f_1,f_1} - \gamma & 0 & \beta_{f_1}\bar{\alpha}_{f_1,f_2} & 0 & \beta_{f_1}\bar{\alpha}_{f_1,m_1} & 0 & \beta_{f_1}\bar{\alpha}_{f_1,m_2} \\ 0 & 0 & -(\mu_{f_2} + \sigma_{f_2}) & -\gamma\overline{N}_{f_2} & 0 & 0 & 0 & 0 \\ 0 & \beta_{f_2}\bar{\alpha}_{f_2,f_1} & 0 & \beta_{f_2}\bar{\alpha}_{f_2,f_2} - \gamma & 0 & \beta_{f_2}\bar{\alpha}_{f_2,m_1} & 0 & \beta_{f_2}\bar{\alpha}_{f_2,m_2} \\ 0 & 0 & 0 & 0 & -\mu_{m_1} & -\gamma\overline{N}_{m_1} & 0 & 0 \\ 0 & \beta_{m_1}\bar{\alpha}_{m_1,f_1} & 0 & \beta_{m_1}\bar{\alpha}_{m_1,f_2} & 0 & \beta_{m_1}\bar{\alpha}_{m_1,m_1} - \gamma & 0 & \beta_{m_1}\bar{\alpha}_{m_1,m_2} \\ 0 & 0 & 0 & 0 & 0 & 0 & -(\mu_{m_2} + \sigma_{m_2}) & -\gamma\overline{N}_{m_2} \\ 0 & \beta_{m_2}\bar{\alpha}_{m_2,f_1} & 0 & \beta_{m_2}\bar{\alpha}_{m_2,f_2} & 0 & \beta_{m_2}\bar{\alpha}_{m_2,m_1} & 0 & \beta_{m_2}\bar{\alpha}_{m_2,m_2} - \gamma \end{bmatrix}$$

where $\bar{\alpha}_{i,j} = (1-\lambda_d)\alpha_{i,j}^{(0)} + \lambda_d\alpha_{i,j}^{(1)}\overline{N}_j$, for $0 \le \lambda_d \le 1$.

$$
\boldsymbol{D}(\bar{X}_{DFPE}) = \begin{bmatrix}
0 & 0 & p\,\nu\,\Psi_1 & 0 & 0 & 0 & p\,\nu\,\Psi_2 & 0 \\
0 & 0 & 0 & \rho & 0 & 0 & 0 & 0 \\
1 & 0 & 1 & 0 & 0 & 0 & 0 & 0 \\
0 & \frac{\bar{N}_{f_1}}{\bar{N}_{f_1} + \bar{N}_{f_2}} & 0 & \frac{\bar{N}_{f_2}}{\bar{N}_{f_1} + \bar{N}_{f_2}} & 0 & 0 & 0 & 0 \\
0 & 0 & (1-p)\,\nu\,\Psi_1 & 0 & 0 & 0 & (1-p)\,\nu\,\Psi_2 & 0 \\
0 & 0 & 0 & \rho & 0 & 0 & 0 & 0 \\
0 & 0 & 0 & 0 & 1 & 0 & 1 & 0 \\
0 & 0 & 0 & 0 & 0 & \frac{\bar{N}_{m_1}}{\bar{N}_{m_1} + \bar{N}_{m_2}} & 0 & \frac{\bar{N}_{m_2}}{\bar{N}_{m_1} + \bar{N}_{m_2}}
\end{bmatrix}
$$

2) Second, we consider the extinction equilibrium ExE solution $\bar{X}_{ExE}(t) = \left(0, \bar{P}_{f_1}(t), 0, \bar{P}_{f_2}(t), 0, \bar{P}_{m_1}(t), 0, \bar{P}_{m_2}(t)\right)^T$ in which not only the population gets extinct, but the disease halts also to spread, since for instance $\bar{I}_{f_1}(t) = \bar{N}_{f_1}(t)\ \bar{P}_{f_1}(t) = 0$ whatever was $\bar{P}_{f_1}(t)$. $\bar{X}_{ExE}$ always exists.

That would result in

$$
\mathbf{J}(\bar{X}_{ExE}) = \begin{bmatrix}
-\mu_{f_1} & 0 & 0 & 0 & 0 & 0 & 0 & 0 \\
\vartheta_{f_1,f_1} & \beta_{f_1}\bar{\alpha}_{f_1,f_1} - \gamma & \vartheta_{f_1,f_2} & \beta_{f_1}\bar{\alpha}_{f_1,f_2} & \vartheta_{f_1,m_1} & \beta_{f_1}\bar{\alpha}_{f_1,m_1} & \vartheta_{f_1,m_2} & \beta_{f_1}\bar{\alpha}_{f_1,m_2} \\
0 & 0 & -(\mu_{f_2} + \sigma_{f_2}) & 0 & 0 & 0 & 0 & 0 \\
\vartheta_{f_2,f_1} & \beta_{f_2}\bar{\alpha}_{f_2,f_1} & \vartheta_{f_2,f_2} & \beta_{f_2}\bar{\alpha}_{f_2,f_2} - \gamma & \vartheta_{f_2,m_1} & \beta_{f_2}\bar{\alpha}_{f_2,m_1} & \vartheta_{f_2,m_2} & \beta_{f_2}\bar{\alpha}_{f_2,m_2} \\
0 & 0 & 0 & 0 & -\mu_{m_1} & 0 & 0 & 0 \\
\vartheta_{m_1,f_1} & \beta_{m_1}\bar{\alpha}_{m_1,f_1} & \vartheta_{m_1,f_2} & \beta_{m_1}\bar{\alpha}_{m_1,f_2} & \vartheta_{m_1,m_1} & \beta_{m_1}\bar{\alpha}_{m_1,m_1} - \gamma & \vartheta_{m_1,m_2} & \beta_{m_1}\bar{\alpha}_{m_1,m_2} \\
0 & 0 & 0 & 0 & 0 & 0 & -(\mu_{m_2} + \sigma_{m_2}) & 0 \\
\vartheta_{m_2,f_1} & \beta_{m_2}\bar{\alpha}_{m_2,f_1} & \vartheta_{m_2,f_2} & \beta_{m_2}\bar{\alpha}_{m_2,f_2} & \vartheta_{m_2,m_1} & \beta_{m_2}\bar{\alpha}_{m_2,m_1} & \vartheta_{m_2,m_2} & \beta_{m_2}\bar{\alpha}_{m_2,m_2} - \gamma
\end{bmatrix}
$$

Also,

$$
\vartheta_{i,j} = (1 - \bar{P}_i)\ \beta_i \lambda_d \alpha_{i,j}^{(1)} \bar{P}_j
$$

and

$$
\bar{\alpha}_{i,j} = (1 - \lambda_d)\alpha_{i,j}^{(0)},
$$

for $0 \leq \lambda_d \leq 1$, and for all $i, j = f_1, f_2, m_1$, and m_2.

And

$$
\boldsymbol{D}(\bar{X}_{ExE}) = \begin{bmatrix} 0 & 0 & 0 & 0 & 0 & 0 & 0 & 0 \\ 0 & 0 & 0 & \rho & 0 & 0 & 0 & 0 \\ 1 & 0 & 1 & 0 & 0 & 0 & 0 & 0 \\ 0 & \frac{\bar{N}_{f_1}}{\bar{N}_{f_1} + \bar{N}_{f_2}} & 0 & \frac{\bar{N}_{f_2}}{\bar{N}_{f_1} + \bar{N}_{f_2}} & 0 & 0 & 0 & 0 \\ 0 & 0 & 0 & 0 & 0 & 0 & 0 & 0 \\ 0 & 0 & 0 & \rho & 0 & 0 & 0 & 0 \\ 0 & 0 & 0 & 0 & 1 & 0 & 1 & 0 \\ 0 & 0 & 0 & 0 & 0 & \frac{\bar{N}_{m_1}}{\bar{N}_{m_1} + \bar{N}_{m_2}} & 0 & \frac{\bar{N}_{m_2}}{\bar{N}_{m_1} + \bar{N}_{m_2}} \end{bmatrix}
$$

References

Belsare, A. V., and Stewart, C. M. 2020. OvCWD: An agent-based modeling framework for informing chronic wasting disease management in white-tailed deer populations. Ecological Solutions and Evidence, 1(1), pp. 1-7, e12017.

Foley, A. M., Hewitt, D. G., DeYoung, C. A., DeYoung, R. W., and Schnupp, M. J. 2016. Modeled Impacts of Chronic Wasting Disease on White-Tailed Deer in a Semi-Arid Environment. PLoS One, 11(10), e0163592.

Freddy, D. J., Baker, D. L., Bartmann, R. M., and Kufeld, R. C. 1993. Deer and elk management analysis guide, 1992–1994. Colorado Division of Wildlife Report.

Jennelle, C.S., Henaux, V., Wasserberg, G., Thiagarajan, B., Rolley, R.E., Samuel, M.D. 2014. Transmission of Chronic Wasting Disease in Wisconsin White-Tailed Deer: Implications for Disease Spread and Management. PLoS One, 9(3), e91043.

Miller, M. W., Williams, E. S., McCarty, C. W., Spraker, T. R., Kreeger, T. J., Larsen, C. T., and Thorne, E. T. 2000. Epizootiology of chronic wasting disease in free-ranging cervids in Colorado and Wyoming. *Journal of wildlife diseases*, *36*(4), 676–690. https://doi.org/10.7589/0090-3558-36.4.676

Oraby T, Vasilyeva O, Krewski D, Lutscher F. (2014) Modeling seasonal behavior changes and disease transmission with application to chronic wasting disease. *J Theor Biol*. 340:50-59.

Potapov, A., Merrill, E., Pybus, M., and Lewis, M. A. 2016. Chronic wasting disease: transmission mechanisms and the possibility of harvest management. PloS One, 11(3), e0151039.

Roberts MG, Kao, RR. (1998) The dynamics of an infectious disease in a population with birth pulse, *Math Biosci* 149:23–36.

TPDW. 2021. 2021 – 2022 Hunting Season Dates — Texas Parks and Wildlife Department (TPDW). Available at: https://tpwd.texas.gov/regulations/outdoor-annual/hunting/general-regulations/2021_2022_hunting_seasons (Accessed: 11/07/2021).

Williams, E.S., Miller, M.W., Kreeger, T.J., Kahn, R.H. and Thorne, E.T. 2002. Chronic wasting disease of deer and elk: a review with recommendations for management. The Journal of wildlife management, pp.551-563.
